# Baseline beliefs, depression, anxiety, and stress in humanities students in the context of the COVID-19 pandemic

**DOI:** 10.1192/j.eurpsy.2022.1240

**Published:** 2022-09-01

**Authors:** D. Ivanov, Y. Aleksandrovich, I. Gorkovaya, V. Averin, V. Titova, V. Rozhdestvenskiy

**Affiliations:** Saint Petersburg State Pediatric Medical University, Department Of Psychosomatics And Psychotherapy, Saint Petersburg, Russian Federation

## Abstract

**Introduction:**

The COVID-19 pandemic can be seen as mental trauma. The concept of baseline beliefs helps to explain the extent to which mental trauma affects individuals.

**Objectives:**

The study aimed to investigate baseline beliefs in humanities students in Russian universities and analyse the relationship between baseline beliefs and emotional reactions.

**Methods:**

Data collection was carried out between May and July 2020 using a Google form that we developed. A total of 92 humanities students participated in the study. The WAS-37 was used to examine baseline beliefs, and the DASS-21 was used to determine depression, anxiety, and stress levels.

**Results:**

We found that the mean values of the scales “Benevolence in the World” (M = 34.8±6.5), “Self-image” (M = 27.2±4.4), “Luck” (M = 32.7±5.7) and “Controlling beliefs” (M = 27.9±4.0) were above the normative mean values for the Russian population and only the values of the scale “Justice” (M = 20.8±3.8) were below these. All components of baseline beliefs had negative associations with depression, anxiety, and stress; only “Benevolence in the World” was associated exclusively with anxiety (r_s_ = -0.223, p < 0.05), and “Justice” with depression (r_s_ = -0.223, p < 0.05).

**Conclusions:**

In a pandemic, the world around them is perceived by humanities students as less fair. Trust in the world, beliefs about the fairness of the world and a positive self-image are correlated with a more favourable emotional state. By this, we support the view that individuals’ implicit beliefs (baseline beliefs) are related to the severity of the traumatic event.

**Disclosure:**

No significant relationships.

